# COVID-19 Pneumonia in Fully Vaccinated Adults during the Dominance of the Omicron Sublineages BA.1.1 and BA.2 in Mexico

**DOI:** 10.3390/medicina58081127

**Published:** 2022-08-19

**Authors:** Efrén Murillo-Zamora, Xóchitl Trujillo, Miguel Huerta, Mónica Riós-Silva, José Guzmán-Esquivel, Jaime Alberto Bricio-Barrios, Oliver Mendoza-Cano, Agustin Lugo-Radillo

**Affiliations:** 1 Departamento de Epidemiología, Unidad de Medicina Familiar No. 19, Instituto Mexicano del Seguro Social, Av. Javier Mina 301, Col. Centro, Colima 28000, Mexico; 2 Facultad de Medicina, Universidad de Colima, Av. Universidad 333, Col. Las Víboras, Colima 28040, Mexico; 3 Centro Universitario de Investigaciones Biomédicas, Universidad de Colima, Av. 25 de julio 965, Col. Villas San Sebastián, Colima 28045, Mexico; 4 Centro Universitario de Investigaciones Biomédicas, Universidad de Colima-CONACyT, Av. 25 de julio 965, Col. Villas de San Sebastián, Colima 28045, Mexico; 5 Unidad de Investigación en Epidemiología Clínica, Hospital General de Zona No. 1, Instituto Mexicano del Seguro Social, Av. Lapislázuli 250, Col. El Haya, Villa de Álvarez, Colima 28984, Mexico; 6 Facultad de Ingeniería Civil, Universidad de Colima, km. 9 Carretera Colima-Coquimatlán, Coquimatlán, Colima 28400, Mexico; 7 CONACYT—Faculty of Medicine and Surgery, Universidad Autónoma Benito Juárez de Oaxaca, Oaxaca 68020, Mexico

**Keywords:** COVID-19 vaccines, adult, pneumonia, risk

## Abstract

*Background and Objectives:* A nationwide retrospective cohort study was conducted to evaluate the factors associated with the risk of laboratory-confirmed coronavirus disease 2019 (COVID-19)-related pneumonia in fully vaccinated adults during the dominance of the Omicron sublineages in Mexico. *Materials and Methods:* Fully COVID-19-vaccinated adults with laboratory-positive illness and symptom onset from April to mid-June 2022 were eligible. We computed the eta-squared (η^2^) to evaluate the effect size of the study sample. The characteristics predicting pneumonia were evaluated through risk ratios (RRs), and the 95% confidence intervals (CIs) were computed through generalized linear models. *Results:* The data from 35,561 participants were evaluated, and the overall risk of pneumonia was 0.5%. In multiple analyses, patients aged ≥ 60 years old were at increased risk of developing pneumonia (vs. 20–39 years old: RR = 1.031, 95% CI = 1.027–1.034). Chronic pulmonary obstructive disease, type 2 diabetes mellitus, arterial hypertension, chronic kidney disease (any stage), and immunosuppression (any cause) were also associated with a higher pneumonia risk. The η^2^ of all the variables included in the multiple models was <0.06. *Conclusions:* Our study suggests that, even when fully COVID-19-vaccinated, older adults and those with chronic conditions were at increased risk of pneumonia during the dominance of the Omicron sublineages BA.1.1 and BA.2.

## 1. Introduction

In Mexico, the vaccination efforts against coronavirus disease 2019 (COVID-19) in the general population started early in 2021. By the first week of June 2022, about 61% of inhabitants had been fully vaccinated [1].

According to genomic sequencing data, the sublineages BA.2 (B.1.1.529.2) and BA.1.1 (also known as BA.1 + R346K of the Omicron variant (B.1.1.529)) were dominating in Mexican territory from April to mid-June 2022 [2]. These strains were identified in 71% and 23% of sequenced cases [2]. Recently published in vitro evidence suggests that the BA.1.1 and BA.2 sublineages are antigenically equidistant from wild-type SARS-CoV-2, and thus, similarly threaten the efficacy of first-generation vaccines [3].

This study aimed to identify the factors associated with the risk of COVID-19-related pneumonia in fully vaccinated adults during the Omicron sublineages BA.1.1 and BA.2 in Mexico.

## 2. Materials and Methods

We performed a nationwide retrospective cohort study in Mexico during the first half of July 2022. The potentially eligible subjects were fully vaccinated (at least two shots from any COVID-19 vaccine) adults (aged 20 years or older) with laboratory-confirmed (reverse transcription–polymerase chain reaction (RT-PCR) or rapid antigen test via nasal swabbing) COVID-19. Patients with illness onset from 1 April to 15 June 2022 were eligible, and they were identified from the records of a normative system for the epidemiological surveillance of respiratory viral diseases of the Mexican Institute of Social Security (with the Spanish acronym IMSS). Subjects with more than 12 months between the date of the second vaccine shot and the date of illness onset were excluded.

A broader description of the employed laboratory methods was published elsewhere [4,5]. The clinical and epidemiological data of interest were retrieved from the audited surveillance system. The main binary (no/yes) outcome was pneumonia, and it was defined by the presence of clinical (cough, dyspnea, and fever) radiographic (ground-glass opacities from computed tomography scan or chest X-ray) findings in patients with laboratory-positive COVID-19 who required hospital admission [6].

Summary statistics were computed, and generalized linear regression models were used to estimate the risk ratios (RRs) and 95% confidence intervals (CIs). We assessed the eta-squared (η^2^) of the multiple models to evaluate the effect size of the study sample. The analytical procedure was performed using the statistical package Stata MP 14.0 (StataCorp; College Station, TX, USA).

## 3. Results

The data from 35,561 patients were analyzed and the overall risk of pneumonia was 0.5% (*n* = 162/35,561). Most of the participants were female (58.4%), and their mean age (±standard deviation) was 38.2 ± 12.9 years (total range: 20 to 90 years). The mortality risk among patients with pneumonia was 27.8% (*n* = 45/162).

The mean interval between the last vaccine shot and the date of symptom onset was 8.3 ± 3.2 months. About 7 out of 10 of the enrolled patients received the Vaxzevria (38.8%; ChAdOx1, AstraZeneca, Cambridge, UK) or BNT162b2 (28.4%; Pfizer-BioNTech, Mainz, Germany) COVID-19 vaccines.

When compared with patients with mild COVID-19 symptoms, those with pneumonia were older (61.4 ± 17.3 vs. 38.1 ± 12.8 years, *p* < 0.001) and had a higher prevalence of obesity (body mass index equal to or higher than 30, 17.3% vs. 9.0%, *p* < 0.001), previously diagnosed chronic pulmonary obstructive disease (7.4% vs. 0.4%, *p* < 0.001), type 2 diabetes mellitus (42.6% vs. 6.1%, *p* < 0.001), arterial hypertension (54.3% vs. 10.3%, *p* < 0.001), chronic kidney disease (any stage; 22.2% vs. 0.5%, *p* < 0.001) and immunosuppression (any cause excepting type 2 diabetes mellitus; 5.6% vs. 0.4%, *p* < 0.001). No significant differences were observed between the study groups in terms gender and tobacco use (current).

In the multiple analysis (Table 1) and when compared with younger subjects (20–39 years old), patients aged 60 years or above were at increased risk of pneumonia (RR = 1.031, 95% CI = 1.027–1.034). The highest increase in pneumonia risk was documented in patients with chronic kidney disease (any stage; RR = 1.146, 95% CI = 1.136–1.156). Type 2 diabetes mellitus, arterial hypertension, and immunosuppression were also associated with an increased risk of developing pneumonia. The η^2^ of all the variables included in the multiple models (Table 1) was <0.06, so the effect size may be considered small–medium.

## 4. Discussion

Our study evaluated the factors predicting COVID-19-related pneumonia in fully vaccinated adults and during the dominance of the Omicron sublineages BA.1.1 and BA.2 in Mexico. We identified populations at risk that may benefit from specific interventions focusing on reducing the transmission of viral respiratory pathogens.

We found that increasing age seems to be an independent risk factor for pneumonia, even in fully immunized adults. However, the risk of severe manifestations among elderly subjects (aged 60 years or above) in our study was 4.2% (*n* = 96/2177), which is considerably lower than the risk observed during the dominance of the wild-type strain and which was as high as 60% [7].

A lower antibody response after vaccination has been documented in older individuals [8]. In addition, the interval between the date of the most recent vaccination and the date of symptom onset was higher among elderly participants. This latter was due to the prioritization of older adults at the start of vaccination efforts in the general population. We consider that these two aspects may be determined, at least partially, by the observed scenario among aged participants.

The association between chronic comorbidities and the risk of pneumonia in COVID-19 has been largely known [9]. In our study sample, the highest risk (RR = 1.146, 95% CI = 1.136–1.156) was documented in a patient with a personal history of chronic kidney disease (any stage), which shows epidemic characteristics in Mexico [10].

According to the most recent local guidelines, molecular testing for COVID-19 is performed only in cases requiring non-ambulatory management. A positive RT-PCR was available for 3.2% (*n* = 1130) of participants (all of them had pneumonia); the remaining analyzed cases were confirmed using antigen-based testing.

We lacked genomic sequencing data for all the analyzed individuals, which represents a significant limitation of the study. However, according to the official data from the General Directorate of Epidemiology of Mexico, the dominance of the two analyzed sublineages was clear, and it agrees with a growing trend in the number of confirmed cases throughout the Mexican territory. In addition, we only analyzed fully vaccinated subjects and, therefore, we were unable to assess the risk of COVID-19-related pneumonia in non-vaccinated adults.

## 5. Conclusions

We characterized the risk of COVID-19-related pneumonia in a large set of fully vaccinated adults during the dominance of the Omicron sublineages BA.1.1 and BA.2. We identified the populations at risk that may benefit from maintaining more strict non-pharmaceutical interventions against COVID-19, even if they are fully vaccinated.

## Figures and Tables

**Table 1:** Predictors of pneumonia in laboratory-confirmed COVID-19 among fully vaccinated adults during the dominance of the Omicron sublineages, Mexico, 2022.

Characteristic	RR (95% CI), p			
	Bivariate Analysis		Multiple Analysis	
Gender				
Female	1.000		1.000	
Male	1.001 (0.999–1.003),	0.090	1.001 (0.999–1.002),	0.152
Age group (years)				
20–39	1.000		1.000	
40–59	1.003 (1.001–1.004),	<0.001	1.001 (0.998–1.002),	0.718
60 or above	1.042 (1.039–1.045),	<0.001	1.031 (1.027–1.034),	<0.001
Months elapsed from the last vaccine shot to illness onset				
<6	1.000		1.000	
6 or above	1.004 (1.002–1.005),	<0.001	1.001 (0.999–1.002),	0.363
*Personal history of:*				
Obesity (BMI of 30 or above)				
No	1.000		1.000	
Yes	1.005 (1.002–1.007),	<0.001	1.001 (0.998–1.003),	0.458
Chronic pulmonary obstructive disease				
No	1.000		1.000	
Yes	1.082 (1.070–1.094),	<0.001	1.049 (1.038–1.060),	<0.001
Type 2 diabetes mellitus				
No	1.000		1.000	
Yes	1.028 (1.026–1.031),	<0.001	1.014 (1.011–1.017),	<0.001
Arterial hypertension				
No	1.000		1.000	
Yes	1.022 (1.019–1.024),	<0.001	1.005 (1.002–1.007),	<0.001
Chronic kidney disease (any stage)				
No	1.000		1.000	
Yes	1.169 (1.158–1.179),	<0.001	1.146 (1.136–1.156),	<0.001
Immunosuppression				
No	1.000		1.000	
Yes	1.059 (1.047–1.070),	<0.001	1.036 (1.026–1.048),	<0.001

Abbreviations: COVID-19—coronavirus disease 2019; RR—risk ratio; CI—confidence interval. Notes: (1) Generalized linear regression models were used to compute the RR and 95% CI; (2) the estimates from the multiple analysis were adjusted by all the variables presented in the table; (3) immunosuppression refers to any cause of the inhibition of the normal immune response, excepting those related to type 2 diabetes mellitus.

## Data Availability

All data can be made available upon request to the corresponding authors.
